# The Multiple Recycling Process of Polypropylene Composites with Glass Fiber in Terms of Grinding Efficiency and Selected Properties of Recirculated Products

**DOI:** 10.3390/polym17192625

**Published:** 2025-09-28

**Authors:** Arkadiusz Kloziński, Paulina Jakubowska, Adam Piasecki, Dorota Czarnecka-Komorowska

**Affiliations:** 1 Institute of Chemical Technology and Engineering, Poznan University of Technology, Berdychowo 4, 60-965 Poznan, Poland; paulina.jakubowska@put.poznan.pl; 2 Institute of Materials Engineering, Poznan University of Technology, Jana Pawła II 24, 60-965 Poznan, Poland; adam.piasecki@put.poznan.pl; 3 Institute of Materials Technology, Poznan University of Technology, Piotrowo 3, 61-138 Poznan, Poland; dorota.czarnecka-komorowska@put.poznan.pl

**Keywords:** polypropylene, glass fiber, polymer composites, recycling of polymer, multiple recycling process, grinding of polymer

## Abstract

This study comprehensively discusses the effect of multiple material recycling (five recycling cycles with the same technological conditions: injection molding → grinding → drying → injection molding → …) of commercial polypropylene-glass fiber composites (PPGF) (PP + 10, 20 and 30 wt.% GF) on the performance of the grinding process and the granulometric characteristics of the obtained regrinds, as well as selected surface, mechanical and thermal properties of the composites. An increase in mass (*E_m_*) and volume (*E_v_*) grinding efficiency was confirmed, along with an increase in GF content in the composite and the number of recycling cycles. Both the GF additive and the number of recycling cycles contributed to the deterioration of the aesthetic qualities of the composites (darkening and reduction in gloss). Slight changes in the surface hardness of the test materials were observed as a function of the number of recycling cycles, from 3 to 4% after five recycling cycles. The adverse effect of multiple recycling on the mechanical and thermal properties of PP and PPGF composites has been confirmed. The occurrence and increase in carbonyl index (*CI*) values, as a function of multiples recycling, was confirmed for a composite containing 20 wt.% GF (*CI* in the range from 0.045 to 0.092) and for PPGF containing 30 wt.% GF (*CI* in the range from 0.193 to 0.272). The effect of multiple material recycling on the glass fiber structure in the tested composites was also investigated using scanning electron microscopy (SEM) and optical microscopy. The issues of grinding and changes in the surface properties of PPGF composites in multiple material recycling processes discussed in this article may constitute a source of practical knowledge that will contribute to increasing the use of this type of secondary composite in industrial plastics processing processes.

## 1. Introduction

In recent years, there has been a noticeable increase in the use of polymer composites. This is associated with the search for new materials with better properties than existing polymers and the possibility of replacing traditional raw materials such as wood, glass, and even metal. Despite the many advantages of using polymer composites, one of the greatest challenges currently faced by the industrial and scientific sectors is their recycling. This results from the increase in composite production and the diversity of their applications. The desire to minimize the negative impact of plastics on the environment is one of the main reasons for intensifying research into the development of various types of recycling processes, including the impact of reprocessing (material recycling) and its frequency on selected functional properties of products made from recycled materials and composite recyclates. Knowledge regarding the possible changes in the quality parameters of secondary polymer materials resulting from their recycling, especially their frequency, can contribute to an increased ratio of recycled material in subsequent product life cycles, which is in line with the current widespread trend of developing circular technologies [1,2].

Fiber-reinforced polymer composites constitute a separate group of engineering plastics [3]. Over the years, many types of fillers and fibers have been added to polyolefins and other polymers in an effort to develop new materials or improve existing ones [4]. There is a wide selection of different types of fibers that serve as reinforcement in composite materials. Depending on their origin, it is possible to distinguish natural and synthetic fibers [5,6,7]. Synthetic fibers such as glass fiber (GF), carbon fiber, and aramid fiber exhibit better strength parameters, durability, and moisture resistance than natural fibers, but they are less environmentally friendly [8,9]. Glass fiber reinforcement is probably the most cost-effective and proven option for reinforcing polymer composites to increase their flexural and tensile strength and modulus of elasticity [4]. Polypropylene (PP) composites reinforced with glass fiber (PPGF) are becoming increasingly popular. These materials are characterized by simple manufacturing methods, excellent mechanical properties compared to pure polypropylene, significant cost savings, and recyclability [10]. The properties of PPGF composites depend mainly on the following variables: fiber content [10,11,12,13], fiber length [10,14,15,16], interaction between the fiber and the matrix [12,17], and matrix properties [18,19]. Glass fiber is also used in hybrid polypropylene composites, in which case natural fibers [20,21,22], inorganic powder fillers [23,24,25] and glass balls [26] are used as additional fillers in addition to GF. One of the main advantages of thermoplastic polymer composites with glass fiber [27,28], including composites based on polypropylene, is their recyclability [10,29]. They can be recycled through different processes such as mechanical, chemical, and thermal [27,30,31]. Mechanical recycling, consisting of the steps of regrinding, regranulation, remelting and reinjection molding, is the most commonly used approach for this type of composites because it is an efficient, high-output and technically and industrially feasible method; it can replace virgin material production and has acceptable costs and the least adverse environmental impacts, comparing to other recycling methods [27]. Industrial practice and numerous research studies confirm that the properties of mechanical recycled polymer materials [32,33,34,35,36] are lower than those of virgin polymers. This is because the chains of macromolecules are damaged and broken after repeated shearing and extrusion of polymeric materials, which reduces the molecular weight, thus adversely affecting their mechanical properties [34,36,37]. In the case of PPGF composites, the mechanical destruction of the glass fiber is the primary factor that determines the deterioration of properties in the material recycling process of the composite. It occurs in the chambers of grinders and plasticizing units of processing machines (extruders, injection molding machines) and progresses with an increase in the number of recycling cycles [37,38,39,40]. However, it should be emphasized that polypropylene composites with glass fiber subjected to several cycles of material recycling still maintain mechanical properties at a higher level than unfilled polypropylene [36,38]. Therefore, secondary PPGF composites are a material with high application potential, which can be used, e.g., for the production of technical products where strength requirements are higher than for unfilled PP, or as reinforcing additives in polypropylene processing. It is therefore justified to conduct research on the process of multiple material recycling of PPGF composites in order to obtain technological information that can increase the potential use of the above-mentioned recyclates in plastic processing.

Most of the studies that analyzed the effect of multiple material recycling on the properties of glass fiber reinforced polypropylene composites focused on changes of selected mechanical [37,38,39,40], thermal [37], and rheological [37,40] properties. One of the reasons for undertaking this research is the lack of reports that describe changes in the surface properties of PPGF composites as a function of the number of processing cycles, which are crucial for achieving appropriate aesthetics and usability of polymer products. The second theme of this highly practical work, which can serve as a source of technological information, is an attempt to analyze the efficiency of the composite grinding process and the granulometric characteristics of the products manufactured in successive grinding cycles. This type of research is significant from the economic perspective of recycling processes in a circular technology, as it takes into account the properties of the intermediate product of material recycling (the regranulate), the process of its production and the properties that determine its further processability. Therefore, the presented article describes the effect of multiple material recycling (five recycling cycles) of polypropylene composites containing 10, 20 and 30 wt.% of glass fiber on the efficiency of the grinding process and the characteristics of the obtained ground material (sieve analysis, bulk density). Surface properties (color, gloss, and hardness), mechanical properties (static tensile and flexural tests, impact strength), and thermal properties (Vicat softening temperature, heat deflection temperature) of products obtained from the milled material after each of the five recycling cycles were also evaluated. Potential changes in the chemical structure of polypropylene, which may result from degradation processes occurring in the composite matrix, were determined based on the carbonyl index assessment using the ATR-FTIR measurement technique.

## 2. Materials and Methods

### 2.1. Materials

Commercial polypropylene composites with glass fiber from Polimarky Sp. z o. o. Sp. K. (Rzeszow, Poland) were used in the study. The first one, called RESLEN PPH3 GF10, contained 10 wt.% glass fiber, while the second one, called RESLEN PPH3 GF30, contained 30 wt.% glass fiber. The composite matrix was homopolypropylene with the trade name TATREN HT 3 06 (produced by SLOVNAFT, a.s., Bratislava, Slovak Republic). A composite containing 20 wt.% glass fiber (GF20) was made by mixing polypropylene and a composite containing 30 wt.% GF at appropriate ratios and homogenization with cold granulation in the twin-screw extrusion process (twin-screw extruder model ZM/258/21, by Zamak Mercator (Skawina, Poland)). E-type glass fiber, type 508A, coated with silane, with a diameter of 13 μm and a length of 4.5 mm (manufactured by JUSHI GROUP CO., LTD., Tong Xiang, China), was the filler for the above-mentioned composites. The designations of polymeric test materials used in the study and their basic properties (density, mass flow rate index) are listed in Table 1.

### 2.2. The Multiple Mechanical Recycling Process

**Figure 1:**
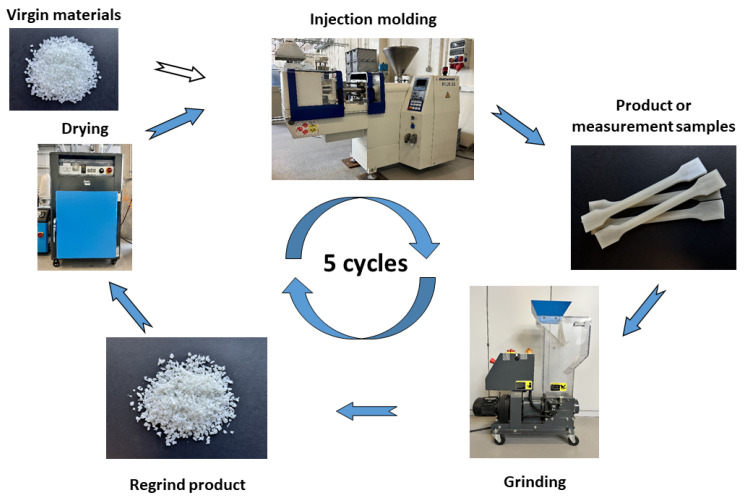
Schematic diagram of the multiple mechanical recycling procedure (closed-loop recycling) used in the study.

The research materials were subjected to a multiple material recycling process using circular technology, according to the scheme shown in Figure 1. Injection molding was the processing technique used in the research. Mechanical recycling (5 cycles) was carried out using a low-speed grinder. The material was dried before each injection process.

The injection process was carried out using a Battenfeld (Kottingbrunn, Austria) PLUS 35 hydraulic injection molding machine and a universal injection mold with interchangeable cavities. The injection parameters were the same for all test materials (PP and PPGF composites containing 10, 20, and 30 wt.% GF) and all processing cycles. The maximum injection temperature was 230 °C, the injection mold temperature was 25 °C, and the cooling time in the mold was 20 s. For each material, dumbbells compliant with ISO 527 and bars compliant with ISO 178 and ISO 179-1 were manufactured and used in the tests. The products in the form of dumbbells, together with the sprues, were subjected to mechanical recycling (five recycling cycles) using a low-speed grinder manufactured by SHINI, model SG-14 (SHINI PLASTICS TECHNOLOGIES, INC., Taipei, Taiwan), with a rotational speed of 26 rpm. The grinder was equipped with two knives (cutting elements with special geometry), equipped with sets of milling teeth and bending-crushing drivers. The theoretical capacity of the used grinder is 3 kg/h. The effect of GF content in composites and recycling rate on the efficiency of the grinding process was determined for 200 g of test dumbbells and weighing the ground material obtained after 10 min of mill operation-the measurement was performed 5 times for each material. The grinding efficiency determined in the above manner was converted into efficiency in g/min. Appropriate abbreviations were introduced for the obtained ground materials, indicating the number of recycling cycles (1–5), e.g., 1PPGF10 represents a polypropylene composite containing 10 wt.% of glass fiber subjected to a single recycling cycle, whereas 5PPGF10 is used for a composite that has been recycled five times. All ground material was dried in a SHINI forced air circulation dryer, model CD5 (SHINI PLASTICS TECHNOLOGIES, INC., Taiwan), at a temperature of 80 °C for 8 h prior to further processing.

### 2.3. Analytical Methods

The sieve analysis of the grinding was performed using a vibrating shaker manufactured by FRITSCH GmbH, model ANALYSETTE 3 SPARTAN (Idar-Oberstein, Germany). The measurements were carried out at an amplitude of 1.5 mm for 5 min, using sieves with mesh sizes of 2.5, 4.0, 5.0, and 7.0 mm. For each of the five samples, 200 g of ground material was used for a given test material. The content of a specific fraction is expressed by wt.%.

All recycled materials obtained in the recycling process were subjected to bulk density ($\rho_{b}$) assessment in accordance with ISO 60 (method R). Bulk density was determined using the following formula:

(1) $$\rho_{b}=\frac{m}{V}\;\left(g/{\mathrm{cm}}^{3}\right)$$

where: *m* is the mass, in grams (g), of the contents of the measuring cylinder; *V* is the volume, in milliliters, of the measuring cylinder (i.e., 100).

The grinding mass contained in the cylinder was determined using an Axis laboratory balance, model BDM6 (Axis Sp. z o.o., Gdansk, Poland).

Sieve analysis and bulk density assessment were performed for the regrinds after each recycling cycle (1–5 cycles).

The effect of GF addition and multiple recycling process on the gloss of PP and PPGF composites was determined using a gloss-meter from TestAn, series DT-268 (Gdansk, Poland). The measurement was carried out in accordance with the ASTM D2457 standard, using a measurement angle of 60°. The surface gloss was quantified according to the specular gloss value in gloss units (GU). The resolution of the glossmeter was 0.1 GU within the range of 0–100 GU.

The hardness of research materials was evaluated using a static Shore hardness tester from Zwick (ZwickRoell GmbH & Co. KG, Ulm, Germany), according to the ISO 868 testing standard. The measurements were performed using the Shore-D scale.

Mechanical properties under static elongation conditions, such as tensile modulus (*E_t_*), tensile strength (*σ_M_*) and elongation at break *ε_B_*, were evaluated for the samples by means of the static tensile test, according to the ISO 527 standard. The tensile tests were performed using a ZwickRoell Z020TH AllroundLine universal testing machine. The traverse speed was set to 1 mm/min during the determination of the *E_t_* and 100 mm/min during the remaining part of the test. Mechanical properties under static flexural conditions, such as the flexural modulus (*E_f_*) and flexural stress at conventional deflection (*σ_fc_*) were evaluated for the samples by means of the static flexural test, according to the ISO 179 standard. The flexural tests were performed using the aforementioned universal testing machine. The traverse speed was set to 2 mm/min during the determination of all flexural mechanical properties.

Charpy impact strength tests were performed on notched specimens using the Instron CEAST Charpy impact testing machine, model 9500 (Norwood, MA, USA). The adopted test method was carried out according to the ISO 179 standard. Impact loading was carried out with a 5.0 J pendulum.

Vicat softening point temperature (*VST*) and heat deflection temperature (*HDT*) investigations were prepared with the use of the CEAST HV3 apparatus (Norwood, MA, USA). Measurements were carried out in an oil bath in accordance with ISO 306 and ISO 75 standards. The *HDT* A type experiment was prepared with the heating rate of 120 °C/h and 1.8 MPa load, while *VST* was determined in the A120 measurement configuration, i.e., a load of 10 N and heating rate of 120 °C/h.

Fourier Transform Infrared Spectroscopy (*FTIR-ATR*) was used to evaluate changes in the chemical structure of polymeric materials as a result of multiple recycling. Infrared spectra were collected using a Nexus Nicolet 5700 Fourier Transform Infrared Spectrophotometer (*FTIR*, Thermo Electron Scientific Instruments Corporation, Madison, WI, USA) equipped with an attenuated total reflection (*ATR*) accessory with a diamond crystal, and measurements were taken at room temperature in the range of 4000–650 cm^−1^, with a resolution of 4 cm^−1^ and 32 scans. The carbonyl index (*CI*) was determined for each of the tested systems in order to conduct a quantitative assessment of polymer degradation. A peak-height-based method was used, according to the following formula [41,42]:

(2) $$CI=\frac{h_{v_{s}}C=O}{h_{\delta_{as}}{CH}_{3}}$$

where $h_{v_{s}}C=O$ is the peak height associated with the carbonyl group, at approx. 1735 cm^−1^ (stretching vibration symmetrical); $h_{\delta_{as}}{CH}_{3}$ is the peak height associated with the methyl group, at approx. 1455–1460 cm^−1^ (deformation vibration asymmetrical).

The effect of recycling on the GF structure was determined using a Nikon Labophot-2 optical microscope equipped with an Optika Optikam Pro 6 camera and a heating stage. The observation was carried out using a 10× magnification lens. The image of the glass fibers was recorded at a temperature of 210 °C (heating rate 40 °C/min) after complete melting of the polymer matrix.

The microstructure of polypropylene and polypropylene composites was analyzed using a SEM microscope MIRA3 (TESCAN GROUP, a.s., Brno, Czech Republic). The carbon coating (approx. 20 nm) was deposited on samples using a JEE 4B vacuum evaporator (JEOL USA, Inc., Peabody, MA, USA).

Measurements of color change, gloss, hardness, assessment of mechanical and thermal properties, structural and microscopic tests were carried out for all test materials after each recycling cycle (1–5 cycles) and for virgin materials (0 cycles). Each of the measurements carried out during the tests was performed five times, and the results presented in the article (tables, figures) represent the average of the five measurements together with the standard deviation value.

## 3. Results and Discussions

### 3.1. Efficiency of the Grinding Process

One of the basic technological processes in the mechanical recycling of plastic products is their fragmentation [43]. Grinding is a common fragmentation technique used in plastics processing [33,44]. The grinding processes results in reduced dimensions of the material, corresponding to the desired size distribution, while increasing the product surface area [45]. Currently, the efficiency of grinding is a notable parameter, particularly with regards to the relationship between efficiency of machine grinding and size reduction of separate grain [46]. Grinding efficiency is very important in terms of the efficiency of plastic processing and recycling processes, but also in terms of energy consumption–it is estimated that up to 8% of global electricity demand is used for grinding, granulation, and agglomeration processes [46]. Polymer materials, including polymer composites, are ground using mills with various designs, and construction as well as mode of operation directly affect their performance [44,45,46,47]. All grinding methods involve overcoming the cohesive forces of the material through mechanical action and require energy consumption, the amount of which is proportional to the strength of the material and the increase in the total surface area of its grains [48]. It can therefore be assumed that the varying GF content in composites and the number of times they are processed may affect the efficiency of the grinding process. As a consequence, an attempt was made to evaluate the efficiency of the grinding process for all test materials during each of the five recycling cycles. The mass grinding efficiency (*E_m_*) was determined as the amount of grinding in grams obtained during 1 min of mill operation (g/min). As shown in Table 1, the addition of glass fibers directly affects the density of the composites produced, causing it to increase with the increase in GF content in polypropylene. In order to obtain a more objective picture of the impact of the GF additive on the grinding process efficiency, the volumetric grinding efficiency (*E_v_*) was also determined, taking into account the specific volume (*v*) of the test materials. The specific volume *v* (p, T) is commonly used in the description of rheological, thermal and mechanical properties of polymers [49,50] and denotes the inverse value of density *d* (p, T). The volumetric grinding efficiency was determined based on the relationship:

(3) $$E_{v}=E_{m}\cdot v\;\left({\mathrm{cm}}^{3}/\min\right)$$

where *E_m_* is the mass grinding efficiency (g/min); *v* is the specific volume (cm^3^/g).

**Figure 2:**
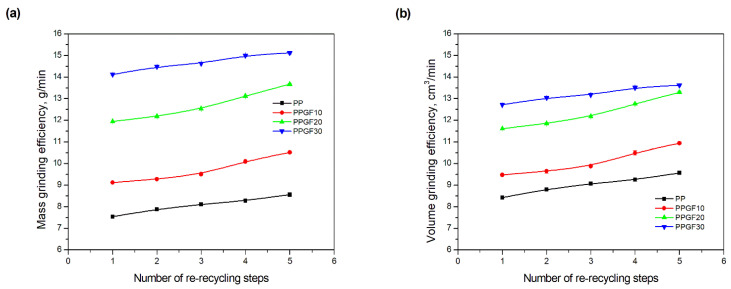
Grinding efficiency of PP and PPGF composites versus number of re-recycle steps: (**a**) mass grinding efficiency; (**b**) volume grinding efficiency.

Grinding was carried out at a constant mill speed and with the same feed form and mass (see Section 2.3). The obtained values of mass and volume grinding efficiency, as a function of the number of re-recycle steps, are shown graphically in Figure 2.

**Figure 3:**
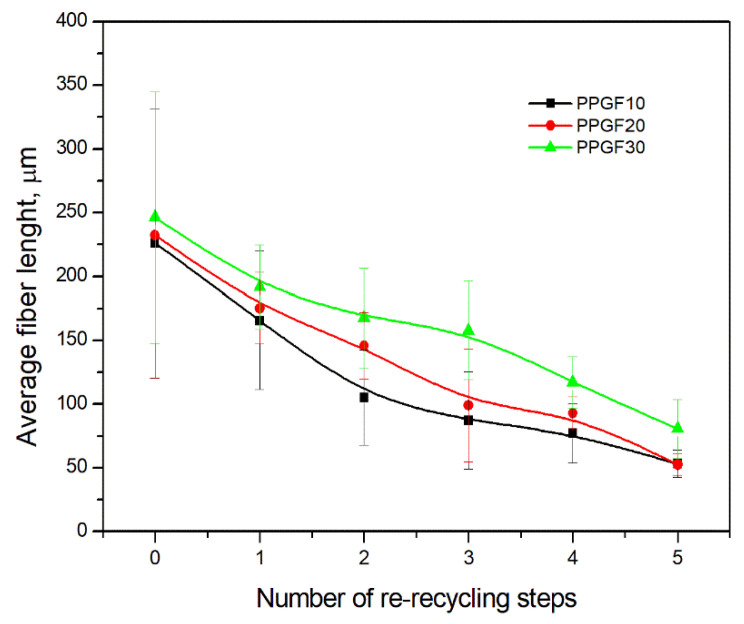
Average fiber length of PPGF composites versus number of re-recycle steps.

As indicated by the relative positions of the curves shown in Figure 2a, the addition of glass fiber to PP increases the mass efficiency of the grinding process. The addition of 10 wt.% GF increased *E_m_* from 7.54 ± 0.036 g/min (1PP) to 9.12 ± 0.033 g/min (1PPGF10). With a GF content of 30 wt.% in the PP matrix, the grinding efficiency of the polymer (understood as process efficiency) increases by as much as 87.3%, i.e., to 14.12 ± 0.048 g/min. The increase in grinding efficiency refers to the quantitative increase in grinding considered in terms of its mass and volume. The shift in the position of the curves representing changes in the volumetric grinding efficiency (Figure 2b), resulting from the varying GF content in the composites, indicates that the increase in grinding efficiency is not solely the result of a change in the density of the composites (see Table 2). It can be concluded that increased grinding efficiency of PPGF composites, along with an increase in filler content, results in increased susceptibility to brittle fracture due to the specific properties of glass fiber. The addition of GF to the polypropylene matrix causes a change in its mechanical properties, which is evident in practice during, e.g., static tensile tests. The brittle fracture leads to a reduction in elongation at break [10,13]. The increase in the material’s susceptibility to brittle fracture directly translates into increased efficiency of multi-edge cutting mills [46]. This type of mill was used in the present study. In addition to glass fiber content, the number of recycling cycles also affects the efficiency of the grinding process. Grinding efficiency increases with the number of recycling cycles. This applies to both unfilled polymers and composites containing varying degrees of glass fiber filling. For example, for a composite containing 20 wt.% glass fiber (1PPGF20), the mass grinding efficiency at one recycling cycle was 11.94 ± 0.041 g/min, while, after five re-recycling steps (5PPGF20) *E_m_* it increased to 13.67 ± 0.042 g/min. The increase in grinding efficiency is the result of the deterioration of the mechanical properties of the test materials due to their degradation as a result of repeated recycling processes [30,31,32,33,34,35,36,37,38], which leads to a reduction in their cohesive forces and, consequently, to lower resistance in grinding processes. From the point of view of the relationships under consideration, the addition of glass fiber has a greater impact on the efficiency of the grinding process than the number of recycling cycles. Changes in the chemical structure of PP and its composites will be discussed in detail later in this article. As mentioned in the introduction, in the case of thermoplastic polymer composites containing glass fiber in their structure, in addition to the above-mentioned degradation of the polymer matrix, mechanical destruction of the fibers also occurs as a result of processing and mechanical recycling (shortening of their length due to damage to their structure) [37,38,39,40]. The reduction in fiber length directly translates into a deterioration of the mechanical properties of the composites [37,38,39,40], which also results in an increase in grinding efficiency. After each recycling cycle, glass fiber weakens the composite as a result of mechanical damage, thereby increasing its susceptibility to fragmentation. In practice, this will lead to a reduced energy consumption and accelerated grinding process of secondary PPGF composites in relation to primary materials. Confirmation of changes in fiber length as a result of five re-recycling steps and six injection cycles (fibers for samples made from pure materials were marked as material 0) is provided by the microscopic images shown in Table 2 and changes in the average fiber length are shown in Figure 3.

The grinding of the polymer materials, its efficiency, and the quality of the resulting ground material directly determine its further use in processing. Grinding equipment is often selected to obtain grind streams that can feed subsequent processing steps without additional treatment (e.g., regranulation). In addition to equipment factors, the granulometric parameters of polymer material grains, such as particle size, shape, and bulk density, determine the efficiency and stability of the plasticizing units of processing machines (extruders, injection molding machines) [51]. Therefore, this study also attempts to assess the impact of re-recycling steps and filler content on the particle size distribution and bulk density of the obtained ground material. Particle size was assessed using sieve analysis. This method is commonly used to assess the quality of grinding products, i.e., to quantitatively determine the ratio of individual grain sizes in a sample of the obtained polymer material [46]. The amount of individual grain fractions was determined as a percentage (wt.%) for a 200 g milling sample. The obtained results are presented in Table 3.

An analysis of the particle sizes contained in Table 3 shows that the number of recycling cycles does not have a significant impact on the regrind particle size distribution. However, differences can be observed between different types of plastics. The largest number of residues with particle sizes below 2.5 mm was generated during the grinding of unfilled PP; the average value for all recycling cycles was 26.2 wt.% of regrind. For composites with the lowest GF content (PPGF10), the average content of the <2.5 mm fraction was 25.1 wt.%; for PPGF20, it decreased to 23.51 wt.%; the lowest content of the above-mentioned fraction was obtained for PPGF30 composites, i.e., 17.4 wt.%. The reduction in the content of the <2.5 mm fraction in the mill, along with an increase in the GF content in the composite, results from the phenomenon of an increasing amount of glass fiber particles remaining in the working chamber of the mill and on the walls of the working chamber, due to their electrification and adhesion. In the case of industrial-scale grinding of PPGF composites, the above phenomenon requires technical solutions in the design of mills to prevent glass dust (from damaged glass fibers) from settling in the working chamber of the device. The same downward trend can be observed for the fraction remaining on a sieve with a mesh diameter of 7 mm—an average of 1.1 wt.% for unfilled polypropylene; 0.77 wt.% for PPGF10; 0.42 wt.% for PPGF20, and 0.24 wt.% for the composite with the highest GF content (PPGF30). The sieve analysis showed that most of the fines after each recycling cycle were characterized by a grain diameter greater than 5 mm but not exceeding 7 mm. The averages from the measurements of residues on a sieve with a hole diameter of 5 mm for all materials ranged from 27.5 to 31.9 wt.%. For sieves with a mesh diameter of 4 mm, the range was from 19.5 to 24.8 wt.%, while, for sieves with a mesh diameter of 2.5 mm, the range was from 21.8 to 25.7 wt.%. In the case of fines remaining on screens with mesh sizes of 2.5, 4, and 5 mm, no upward or downward trend was observed for the fractions retained on any of the above-mentioned screens as a function of the amount of GF in the polypropylene matrix. Based on the data shown in Table 3 it can be concluded that the obtained ground materials are characterized by a similar particle size distribution for the fraction with the highest percentage content, regardless of the number of recycling cycles and the percentage of glass fiber in the composite, also in relation to unfilled PP. Therefore, it can be concluded that their processability will be at a comparable level from the perspective of the granulometric characteristic of the regrind particle size (feeding by screw plasticizing systems).

**Figure 4:**
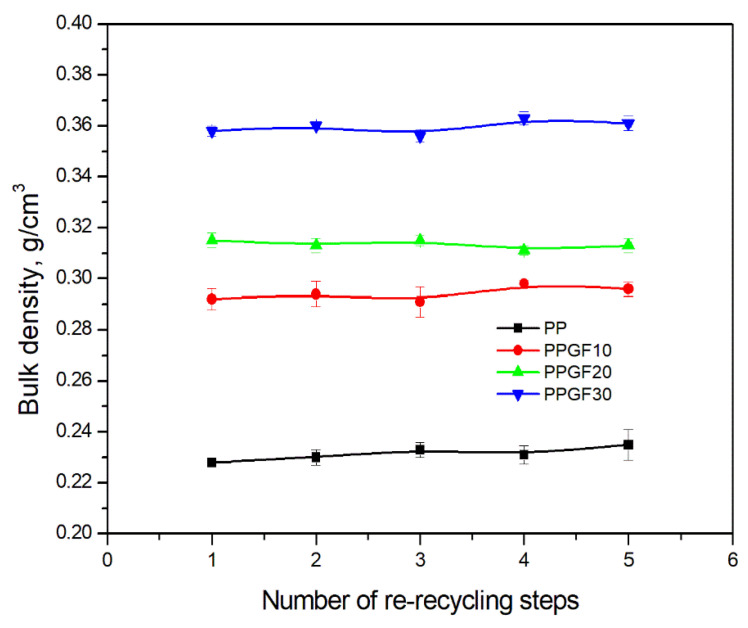
Bulk density of PP and PPGF composites versus number of re-recycle steps.

Bulk density was the second parameter used to characterize the granulometric properties of the research materials determined in this study. This value is important for the geometric characteristics of hoppers in processing equipment (extruders, injection molding machines) and influences the loads on the hopper walls and outlet openings. First and foremost, $\rho_{b}$ determines the degree of filling of the screw channel in the plasticizing system, in the feed opening section, and in part of the feed section, which translates into process efficiency [51]. The bulk density of all regrinds was determined based on Equation (1), in accordance with the procedures of ISO 60 (method R). The measurement results are presented graphically in Figure 4. When comparing the bulk densities of the produced regrinds to pure granules (Table 1), a very significant decrease in the $\rho_{b}$ values obtained in the recycling process of secondary materials can be observed. This is a consequence of the change in the form of the raw materials used from spherical pellets (pure PP) and cylindrical pellets (pure composites) to regrinds with irregular shapes [52,53]. For unfilled polymer, a decrease in bulk density by ~59% is observed, i.e., from 0.550 ± 0.004 g/cm^3^ (0PP) to 0.228 ± 0.003 g/cm^3^ (1PP). For a composite with 10 wt.% GF content, the decrease in $\rho_{b}$ was ~40%, for PPGF20 it was approx. 37%, and, for the highest GF content in polypropylene (PPGF30), it was approx. 27%. Due to the specific nature of their operation, the efficiency of screw plasticizing systems decreases with a decrease in the bulk density of the polymer raw material feeding the process [51,52,53]. Therefore, regrinds, as irregularly shaped products produced in the mechanical recycling process, are characterized by lower efficiency in feeding plasticizing units compared to original raw materials with regular shapes. The growing environmental awareness and the emphasis on the development of circular technologies, including the increased ratio of recycled materials in plastic processing, contribute to the development of research into the design of devices (plasticizing units) that can process them efficiently [52,53]. As indicated by the relationship shown in Figure 4, the number of processing cycles (for the assumed five re-recycling steps) does not affect the bulk density of the obtained regrinds. The shift in the relative positions of the curves shows a clear effect of the GF addition on the $\rho_{b}$ of the composites. The bulk density of the regrinds increases with increasing filler content in the polymer matrix, which, in practice, will lead to increased efficiency of processing processes fed with recyclate with higher amounts of GF fillings.

### 3.2. Surface Properties

**Figure 5:**
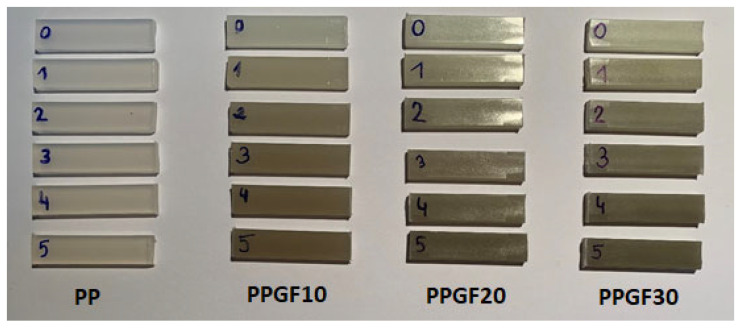
Color of PP samples and PPGF composites after successive re-recycling steps (cycle numbers: 0–5).

The aging processes of polymer materials cause structural changes under the influence of external factors, which affect the deterioration of their performance properties. Polymers and polymer composites can undergo aging during processing, storage, or use [54]. In the case of the presented research, the polymer material subjected to multiple recycling processes in a circular technology is exposed to degradation caused by mechanical factors during processing in the presence of oxygen. Under these conditions, the polymer may undergo thermal degradation (influence of temperature), thermo-mechanical degradation (influence of temperature and mechanical stress), and thermal-oxidative degradation (influence of temperature and oxygen). The degree of degradation occurring in processing is determined by the above-mentioned factors as well as material factors such as modifying additives, fillers, and the chemical structure of the polymer [55,56]. One of the earliest noticeable symptoms indicating the occurrence of aging processes in polymers is a change in their appearance, manifested by a change in color (most often yellowing), loss of gloss, and fading [57]. The process of changes in the appearance of polymer surfaces as a result of aging occurs most intensively as a result of atmospheric aging, but it is also the result of processing degradation [55,57]. Due to the lack of information regarding the impact of multiple recycling processes on the surface properties of polypropylene composites with glass fiber, this study attempts to assess the impact of the re-recycling steps on color and gloss changes. The change in color was determined based on the CIELab mathematical model, which allows the difference in color of the material to be described numerically in relation to a standard [58,59,60]. It should also be emphasized that the recycling process was carried out in accordance with the standards characteristic for material recycling in a shortened circular technology [31] (injection molding → grinding → drying → injection molding, etc.); hence, the material was not exposed to external contaminants that could cause discoloration. The adverse effect of re-recycling on the color change of PP and PPGF composites was also visible to the naked eye (as a result of organoleptic visual assessment). Images of samples showing color changes in the test materials (samples obtained from measuring bars) are presented in Figure 5.

**Figure 6:**
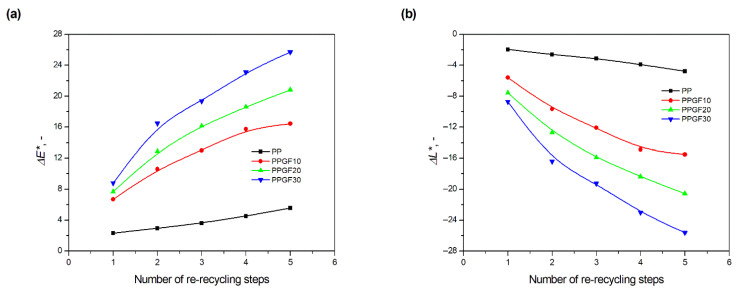
Parameter ∆E* determining to the difference between color (**a**) and parameter ∆L* determining to the different between luminance (**b**) of PP samples and PPGF composites after successive re-recycling steps (cycle numbers: 1–5).

When attempting to describe color changes based on visual assessment, it should first be noted that the color change in polypropylene is caused by the very fact of adding GF to the polymer matrix, and this change intensifies with increasing filler content in PP. The reference sample for measuring the color change in the polymer matrix as a result of the addition of GF was pure PP. The addition of 10 wt.% glass fiber caused a change in color shades, i.e., a ∆E* parameter of 3.65; for 20 wt.% GF addition; the change increased to 7.41, and, at the highest filler content (30 wt.% GF), ∆E* reached a value of 9.32. Changes in luminance value (∆L*), i.e., the value determining the brightness of a sample ranging from 0 (perfectly black sample) to 100 (sample perfectly reflecting incident radiation), resulting from the addition of GF, were as follows: −1.12 for 0PPGF10, −6.51 for 0PPGF20, and −8.84 for 0PPGF30. The recorded changes in the ∆L* parameter confirm the darkening of composites as a function of the increase in the amount of GF in the polymer matrix. As indicated in the images of the samples shown in Figure 5, the change in their color (darkening) increases with the number of processing cycles for unfilled PP and composites. It can also be observed that the darkening effect is more intense for composite samples and increases with the GF content in the composite. The organoleptic observations are confirmed by the coefficient values of the ∆E* and ∆L* determined on the CIELab scale. Their changes as a function of processing multiples are shown graphically in Figure 6. In this case, samples made from pure materials (0PP, 0PPGF10, 0PPGF20, and 0PPGF30) were used as the reference standard in the measurement of color change.

A change in color as a function of the re-recycling steps was observed instrumentally after the first recycling cycle. For the unfilled polymer, the ∆E* parameter changed by 2.31, which, according to literature data, should be visible to the human eye (∆E* > 1) [60]. The parameter that confirmed the change in sample brightness (after 1 recycling cycle) took the value −1.95, which indicates the darkening of the sample. In comparison, the luminescence (L*) of the reference sample was at 64.73. The ∆E* change of polypropylene increased as a function of the number of processing cycles, reaching 5.56 after five recycling cycles. The total change in PP brightness for five re-recycling cycles (manifested in practice by its darkening), represented by the variability in the ∆L* parameter, was −4.76. The position and course of the curves in Figure 6 indicate that greater changes in the ∆E* and ∆L* parameters occurred during multiple processing of composites, and their values increased with the increase in filler content in the polymer matrix. Thus, it can be concluded that the presence of GF promotes the degradation changes occurring under processing conditions, which manifest themselves in a negative impact on the color of the product. Referring the above-mentioned observations to selected numerical data, they were presented as follows. The first recycling cycle of the PPGF10 composite resulted in a ∆E* change to 6.59 and a darkening of the material corresponding to a ∆L* change to −5.59. The total change in color shades for the composite with the lowest GF filling level (10 wt.% GF) after five recycling cycles was 16.44. This was accompanied by darkening corresponding to a decrease in the ∆L* parameter to −15.51. The degree of darkening of the composite containing 20 wt.% GF was even greater. The change in the ∆L* parameter after five cycles of PPGF20 recycling was −20.57, with a total change in color shades to 20.82. The material that exhibited the highest susceptibility to color change as a result of repeated material recycling was the composite with the highest GF filler content. For PPGF30, the ∆L* change after the first recycling cycle was −8.73: the luminescence of the reference sample, i.e., the 0PPGF30 composite, was 63.51. This composite (5PPGF30) underwent the greatest darkening as a result of five cycles of recycling out of all the test materials, i.e., a ∆L* change corresponding to a value of −25.61.

**Figure 7:**
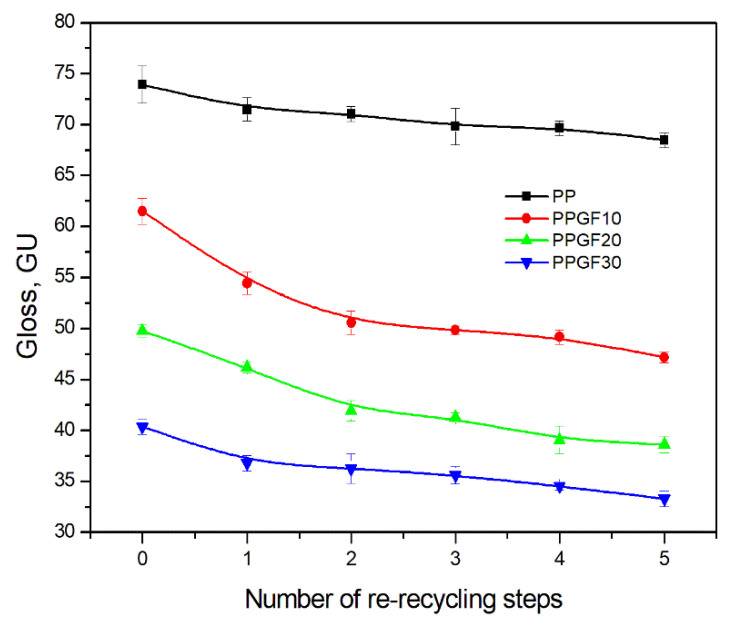
Gloss of PP samples and PPGF composites after successive re-recycling steps (cycle numbers: 0–5).

Multiple material recycling also affected the second most important factor in terms of the aesthetic properties and surface quality of polymer products, namely, gloss. Gloss is an optical impression created by the reflection and scattering of light on the surface of solids and liquids or directly above the surface. It is also defined as the amount of light reflected at the same angle as the light falling on the surface being examined. The gloss value depends, among other things, on the refractive index and absorption coefficient, the transparency and color of the product, the type of lighting, its surface (including structure, roughness, and position of the tested plane), and the angle and distance of observation [60,61]. The gloss of polymer products is determined by the condition of their surface, which is directly related to the processing conditions (including mold temperature and the roughness of the processing tools) [60,61,62], as well as various types of modifiers, dyes, and fillers [61,63,64,65,66]. The gloss of polymer surfaces is also affected by recycling, which deteriorates it as a function of the number of processing cycles, due to degradation processes occurring in the material [67]. The changes in the gloss of the test materials as a function of re-recycling steps are presented in Figure 7. The graph also includes the gloss of molded parts made from pure materials. The data in the chart below show that the surface of PP and PPGF composites loses its aesthetic value, i.e., gloss, as the number of recycling cycles increases. The addition of GF causes the GU to shift towards lower values and changes the classification of composites in terms of their gloss compared to unfilled polymers. According to the literature [68], a surface can be classified as glossy if its GU value is between 51 and 80. For samples made of pure polypropylene, the GU value was 73.95 ± 1.84. The greatest decrease in gloss for PP occurred after one recycling cycle and amounted to approximately 3.3%. Subsequent recycling processes resulted in a decrease in gloss by approximately 0.5 GU per cycle. After the fifth processing cycle, the gloss value decreased to 68.5 ± 0.72 GU, and the material still had a glossy surface. The addition of 10 wt.% GF contributed to a significant reduction in GU compared to the PP matrix; the gloss value for the 0PPGF10 composite material was 61.50 ± 1.31. As in the case of unfilled PP, the greatest decrease in the gloss value for the PPGF10 composite occurred after one recycling cycle: approximately 11.5% (the addition of glass fiber intensified the change in GU as a result of recycling, in relation to the change accompanying one recycling cycle of polypropylene). The third recycling cycle of the composite containing 10 wt.% GF marks the transition point of gloss to a GU value corresponding to a semi-gloss surface (GU = 31–50) [68]. The GU value of the 3PPGF10 composite (i.e., after three re-recycling cycles), the PPGF20 composite, and the PPGF30 composite remained within the above-mentioned range throughout the entire range of re-recycling cycles considered (from zero to five re-recycling steps). The surface gloss of the PPGF10 composite, as a result of five re-recycling cycles (5PPGF10), decreased by approximately 23%, i.e., to a level of GU = 47.22 ± 0.51. For composites with 20 wt.% glass fiber, this change was approximately 22%, while, for PPGF30 composites, the decrease in the GU value was approximately 18%. Based on the above-mentioned analysis of changes in GU values, the processing ratio and glass fiber content significantly affect the gloss of the analyzed test materials, causing it to deteriorate. In industrial practice, this will require additional modifications to recyclates in order to improve their aesthetics in terms of surface gloss.

**Figure 8:**
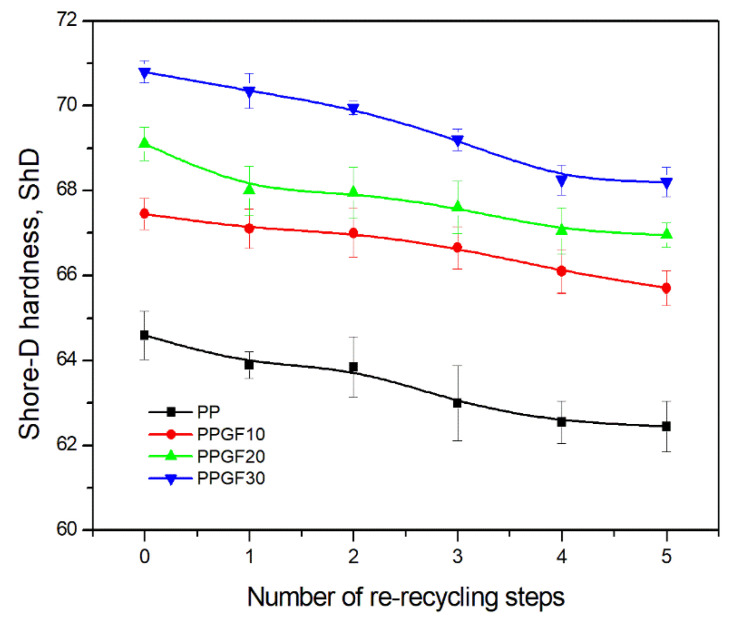
Shore-D hardness of PP samples and PPGF composites after successive re-recycling steps (cycle numbers: 0–5).

The hardness of polymer materials is important from the perspective of surface durability. It can be concluded that the degradation processes of polypropylene and its composites resulting from repeated recycling will cause changes in their structure, which will affect the above-mentioned property [31]. Therefore, this study also assessed the impact of GF additives and the number of recycling cycles on the hardness of PP and PPGF composites. The changes in the obtained Shore-D hardness values are presented graphically in Figure 8. As shown in the relationships below, hardness is influenced by both the glass fiber content in the composite and the number of re-recycling cycles. An increasing concentration of glass fiber in PP causes the hardness to shift towards higher ShD values. This is because the used filler (GF) has better mechanical properties than the used polymer matrix. Glass fiber is a reinforcement that strengthens the composite, and its purpose is to absorb all stresses. As can be seen from the observed changes in hardness, this also applies to the interactions/stresses arising on the surface of the composite. The changes noted confirm the observations described in [69], where it was shown that the addition of glass fiber (10, 20, and 30 wt.% GF) improves the hardness of PP. In the case of the glass fibers used in the tests, the improvement in PP hardness after the addition of 10 wt.% was ~4.4%, i.e., from 64.6 ± 0.6 ShD to 67.45 ± 0.4 ShD. Subsequent amounts of GF additive increased the hardness of the composite by approximately 2.5%. The composite containing 30 wt.% filler (0PPGF30) exhibited the highest hardness, which was 70.8 ± 0.3 Sh^o^. The recycling process did not cause any sudden changes in the hardness of PP and its composites. For all test materials, the decrease in hardness between the hardest material (virgin materials) and the recycled material after five recycling cycles ranged from 3 to 4%. However, attention should be paid to the hardness of PPGF20 and PPGF30 composites after four and five recycling cycles. The degradation changes in the composites after these recycling cycles were so significant that they achieved a lower hardness than the original composite, with a lower degree of glass fiber filling. For comparison, the hardness of 0PPGF20 was 69.1 ± 0.4 ShD, while that of the 4PPGF30 composite decreased to 68.25 ± 0.4 ShD. The addition of 10 wt.% GF caused such an increase in the surface hardness of PP that after none of the five recycling cycles was the hardness of the PPGF10 composite lower than that of unfilled polypropylene.

**Figure 9:**
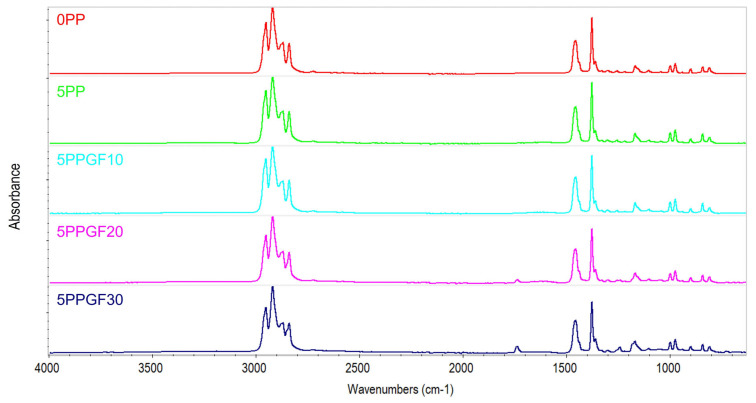
*FTIR-ATR* spectra of virgin polypropylene 0PP, polypropylene 5PP and polypropylene composites PPGF (5PPGF10, 5PPGF20 and 5PPGF30), after five successive re-recycling steps.

During the course of the research, an attempt was also made to assess the degradation changes that occurred in the structure of polypropylene, based on the analysis of infrared spectra obtained using the *FTIR-ATR* measurement technique. The *FTIR* spectra of pure PP (0PP), polypropylene after five recycling cycles (5PP), and composites after five recycling cycles (5PPGF10, 5PPGF20, and 5PPGF30) are presented in Figure 9. The spectra for 0PP show IR bands characteristic of polypropylene spectra, consistent with literature data (Table 4).

**Figure 10:**
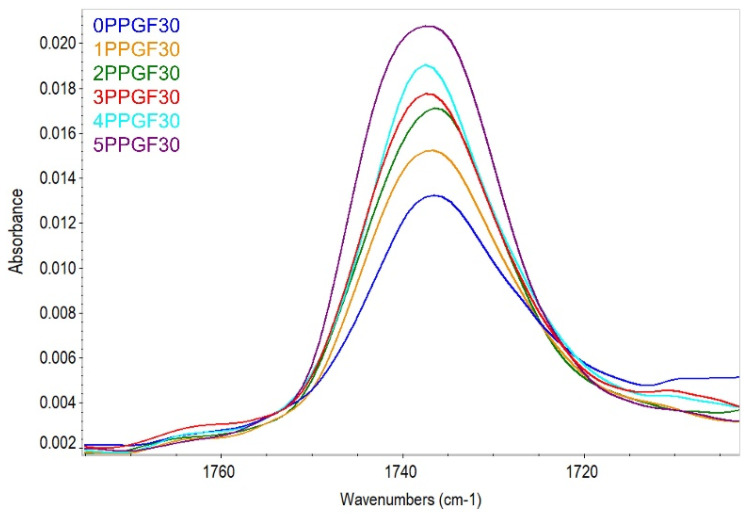
*FTIR-ATR* spectra in the wavenumber range of 1790–1705 cm^−1^, i.e., in the range of the C=O bond absorption band for polypropylene composites PPGF30, after successive re-recycling steps (cycle numbers: 0–5).

**Figure 11:**
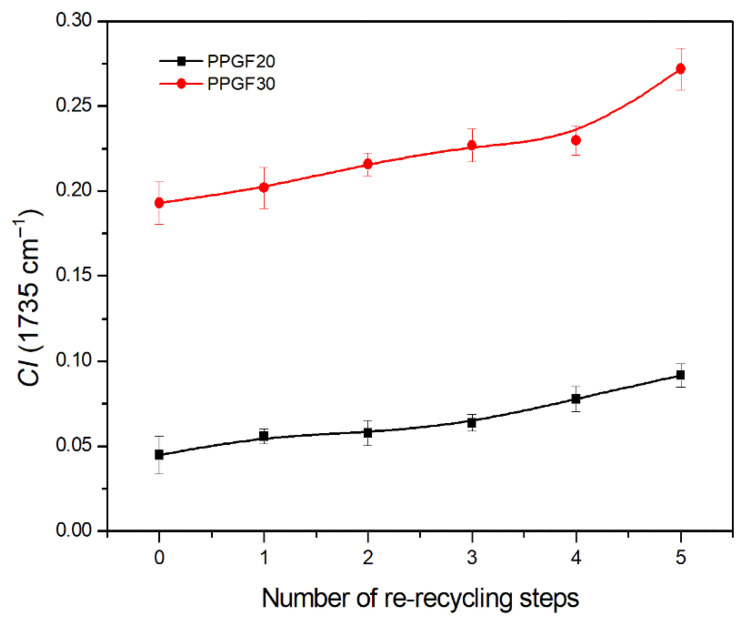
The changes in carbonyl index (*CI*) of PPGF20 and PPGF30 composites after successive re-recycling steps (cycle numbers: 0–5).

During PP degradation, C-C and C-H bonds in the macromolecular chain break, with the simultaneous formation of carbonyl, peroxide, and hydroxyl groups [56]. The spectra of test materials subjected to multiple recycling processes (from zero to five re-recycling steps) did not show bands originating from –OH groups (range: 3700–3200 cm^−1^). The band originating from carbonyl groups appeared only for spectra determined for composites containing 20 and 30 wt.% GF additive. No C=O band was observed for PP and PPGF10 composites after any of the subsequent recycling cycles. As a result of the analysis of the spectra of PPGF20 and PPGF30 composites, a slight increase in the intensity of the peak in the region of 1735 cm^−1^, originating from the C=O group, was observed as the number of recycling cycles increased. Changes in the intensity of the peak originating from the carbonyl group for PPGF30 composites are shown graphically in Figure 10. Using the procedure described in the literature [41,42] and equation (2), the carbonyl index (*CI*) values were determined for PPGF20 and PPGF30 composites. Changes in *CI* values for the above-mentioned composites, as a function of the re-recycling steps, are presented graphically in Figure 11.

Among the tested materials, only two composites with the highest degree of glass fiber filling showed peaks originating from carbonyl. The determined *CI* took very low numerical values, which indicates a low degree of polymer degradation resulting from its oxidation. The numerical values of the carbonyl index in accelerated PP aging processes (250–1000 h), with free access to atmospheric oxygen (photo-oxidative degradation), range from 0.4 to 0.9 [41]. For the PPGF20 composite, the *CI* value ranged from 0.045 to 0.092; for the PPGF30 composite, it ranged from 0.193 to 0.272. In both cases, the index value increased with the number of recycling cycles, indicating the progression of degradation processes in the polymer. Assuming that the kinetics of chain oxidation depend mainly on the diffusion of oxygen into macromolecules, the process was limited under the processing conditions (injection molding). It can therefore be concluded that, in this case, thermal–oxidative degradation was the dominant degradation process, which was facilitated by thermo-mechanical degradation (more intense for materials with a higher GF content: 20 and 30 wt.%). It can also be concluded that oxodegradation processes in the adopted closed material recycling cycle did not occur during the grinding process, as low *CI* values were also found in composite samples made of primary materials (0PPGF20 and 0PPGF30). In their case, the oxidation of PP macromolecules must have occurred during the filling of the GF polymer matrix under industrial conditions or during the first injection process. According to the analysis, PP degradation involving oxidation of its chain affected only two composites. However, degradation processes resulting from other known mechanisms [57] occurred in all test materials, as confirmed by the discussed changes in surface properties. Obtaining a more complete picture of the degradation processes that occur in the polymer matrix of the tested composites would require the extension of measurement techniques, e.g., with DSC measurements or assessment of changes in the molecular weight of PP, which will be the subject of further research. Degradation changes in the material, which intensify with increasing processing frequency, are also manifested in the deterioration of the mechanical and thermal properties of PPGF composites, which will be discussed later in this article.

### 3.3. Mechanical Properties

**Figure 12:**
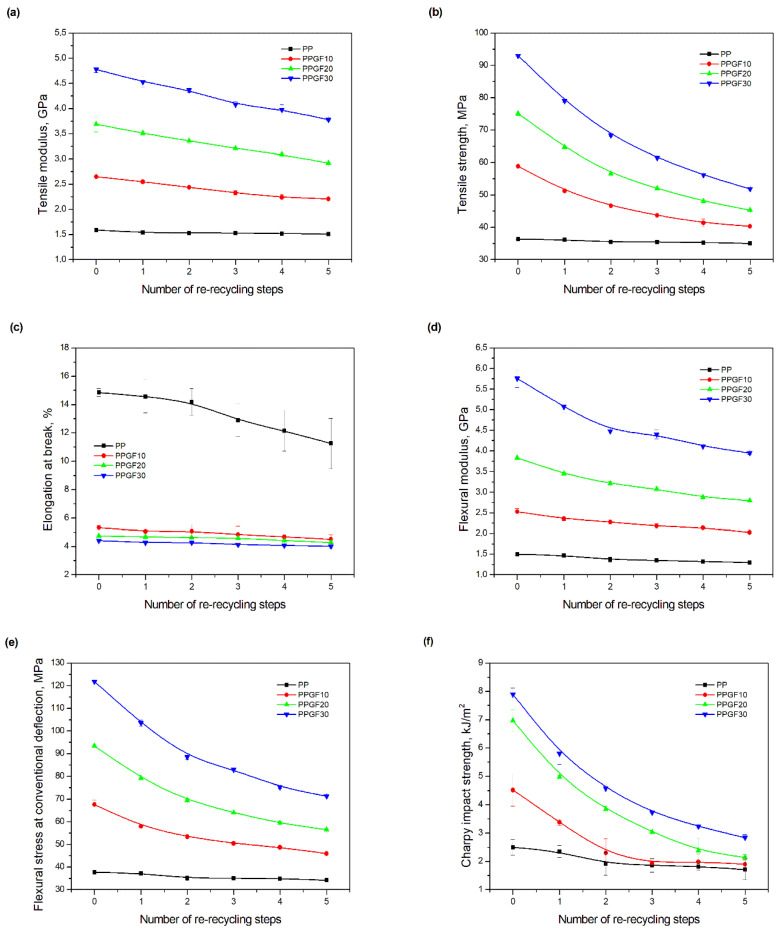
Mechanical properties of PP samples and PPGF composites after successive re-recycling steps, (**a**) tensile modulus; (**b**) tensile strength; (**c**) elongation at break; (**d**) flexural modulus; (**e**) flexural stress at conventional deflection; (**f**) Charpy impact strength.

The main purpose of adding glass fibers to a polymer matrix is to reinforce it. As mentioned in the introduction section, glass fiber reinforcement is the most economical and industrially proven option for reinforcing polymer composites, increasing their strength under static tensile and flexural conditions [4]. This is confirmed by the results of strength tests carried out for PP and PP/GF composites, presented in graphical form in Figure 12. For the original polymer materials, the expected increase in modulus values (tensile modulus (Figure 12a), flexural modulus (Figure 12d)) and strength (tensile strength (Figure 12b), flexural stress at conventional deflection (Figure 12e)), along with an increase in GF content in the polypropylene matrix, was observed. The addition of glass fiber also causes a decrease in elongation at break (Figure 12c) and an increase in PP impact resistance, determined during the tests using Charpy impact strength (Figure 12f). The observed increase in Charpy impact strength with a notch is consistent with literature data, which indicate an improvement in impact resistance up to 40 wt.% glass fiber reinforcement of PP; higher GF content in the composite results in a decrease in its impact strength [16]. For the majority of the considered mechanical properties, proportional changes are observed with an increase in GF content in the composite. The exception is elongation at break, in case of which the highest decrease in value occurs after the addition of 10 wt.% filler. The unfilled polymer exhibited an elongation at break of 14.85 ± 0.28% (0PP), while, for the composite containing 10 wt.% GF (0PPGF10), the elongation decreased to 5.33 ± 0.14%—a change of approximately 64%. The lowest elongation at break was observed in the composite with the highest filler content (0PPGF30), as it amounted to 4.42 ± 0.09%.

Multiple material processing of polyolefins causes changes in their molecular structure. High temperatures, oxidation, and mechanical shearing cause the polymer macromolecules to break down, resulting in their degradation. Any type of interaction in processing, including cutting and grinding, can have a degrading effect on polymer macromolecules, impairing their properties [31]. When analyzing changes in the mechanical properties of polypropylene as a function of the re-recycling steps, it can be clearly stated that five cycles of recycling do not significantly impair these properties. The tensile modulus of the original 0PP was equal to 1.59 ± 0.03 GPa, decreasing most significantly after the first recycling cycle by approximately 3%. The total change in *E_t_* was approximately 5%, reaching a value of 1.51 ± 0.02 GPa for 5PP. Even smaller changes were observed for tensile strength. In this case, fifth-cycle recycling reduced the strength of the polymer by only approximately 3.7%. The greatest impact of degradation processes occurring as a result of repeated recycling of virgin PP was observed for elongation at break. According to literature data [71,72], this parameter is most sensitive to changes in the molecular weight of polymers (its reduction), which occur as a result of degradation processes accompanying material recycling. As a result, a decrease in elongation at break is observed with an increase in the number of recycling cycles. For the polypropylene used in the tests, a decrease in elongation at break by approximately 24% was observed as a result of fifth-cycle recycling, i.e., from 14.85 ± 0.28% to 11.25 ± 1.78%. The adverse changes occurring in PP are also reflected by the low repeatability of elongation results during stretching—an upward trend in the standard deviation of measurements can be observed with increasing recycling frequency. The degradation changes that occurred as a result of repeated recycling in PP are also visible in its response to flexural loads. The flexural modulus and flexural stress at conventional deflection decrease with increasing number of recycling cycles, by approximately 13% *E_f_* and approximately 9% *σ_fc_* for each subsequent recycling cycle.

**Figure 13:**
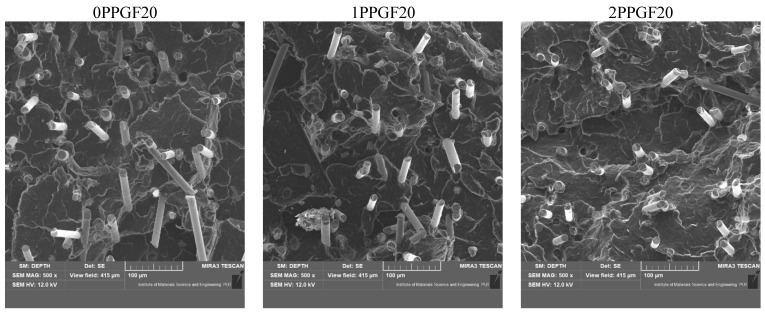

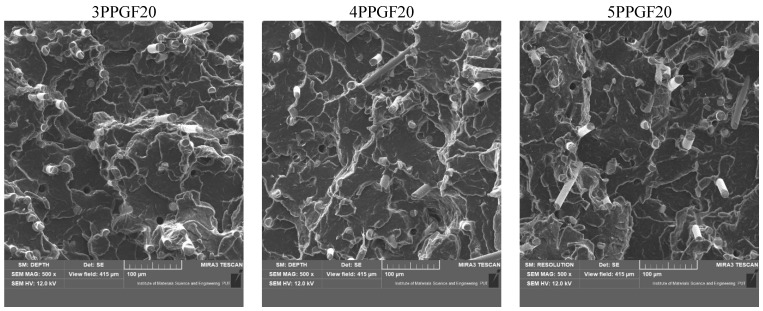
SEM micrographs of PPGF20 composites after successive re-recycling steps (1–5).

The deterioration of the mechanical properties of polypropylene composites with glass fiber as a result of multiple recycling processes consists of degradation processes of the polymer matrix and mechanical destruction of the glass fiber (destruction of the structure, shortening of the fibers) which occur in the operating chambers of processing equipment [37,38,39,40]. The mechanical properties of a composite are determined by the strength of the polymer matrix, the strength of the fibers, and, to a greater extent, the fiber content, fiber orientation, and fiber shape (diameter, length) [10,11,12,13,14,15,16,17,18,19]. Increasing the GF content in the composite is the fastest way to improve its strength [10,12,13,14,15,16,17,19], which was also confirmed by the results of this study. With the same glass fiber content, polypropylene composites containing longer fibers exhibit more favorable mechanical properties. However, a decrease in GF length leads to a reduction in its strength parameters [14,15]. A decrease in fiber length is generated at each reprocessing step [33], resulting in a gradual deterioration of its mechanical properties as a function of the number of recycling cycles [33,37,38,39]. Confirmation of changes in GF length (its reduction) after successive re-recycling stages is provided by images of fiber morphology presented in Table 2; the data in Figure 3 show the morphology of composite sample fractures imaged using scanning electron microscopy (SEM). An example of the morphology of fractures in composites containing 20 wt.% glass fiber (PPGF20), obtained using SEM measurement technique, is shown in Figure 13.

During the analysis of changes in the mechanical properties of composites, it was observed that the deterioration in the strength parameters as a function of the re-recycling steps is favored by an increase in the GF content in the composite. Comparison of the changes in tensile strength after five recycling cycles leads to the following results: 31.6% for PPGF10, 39.7% for PPGF20, and 44.2% for PPGF30. In the case of the flexural modulus, the decrease in its value was equal to 19.8% for PPGF10, 26.9% for PPGF20, and 31.4% for PPGF30, respectively. This relationship applies to all considered mechanical properties. It can be concluded that this is a consequence of the increased degree of mechanical destruction of GF as a function of the number of recycling cycles, along with an increase in its content in the polymer matrix. Additionally, an increase in polymer degradation can be observed as a result of intensified mechanical interactions/mechanical stresses between the polymer and glass fiber in the plasticizing system of the injection molding machine, along with an increase in GF content in the composite. Confirmation of the increased degree of degradation of PP composites with a higher GF content is provided by e.g., the changes in *CI* shown in Figure 11 and discussed earlier in the article (Section 3.2). None of the composites, in all the considered recycling cycles (with the exception of impact strength and elongation at break), exhibited worse mechanical properties compared to unfilled PP. Therefore, even after five recycling cycles, these materials can be a potential reinforcing raw material for pure polypropylene.

During the production of parts in the injection molding process, the fibers are oriented in specific directions due to shear and elongation stresses in the thermoplastic melt depending on the mold-related flow processes [73]. This fiber orientation has a direct influence on the mechanical properties and the anisotropic behavior of the composites, since the composites exhibit significantly higher mechanical properties when loaded in the fiber direction due to the higher characteristic values of the fibers [73,74,75]. Changes in the length of glass fibers and degradation of polypropylene resulting from repeated mechanical recycling and injection molding can directly translate into differences in the arrangement and orientation of GF in molded parts obtained in subsequent production cycles. Therefore, this issue will be a potential topic for further research conducted by the authors in the field of multiple recycling of PPGF composites.

### 3.4. Thermal Properties

**Figure 14:**
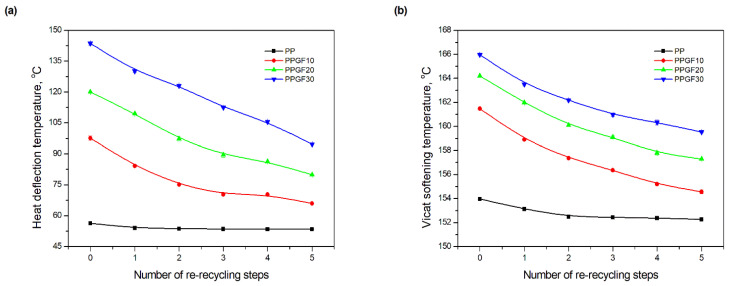
Thermal properties of PP samples and PPGF composites after successive re-recycling steps: (**a**) heat deflection temperature; (**b**) Vicat softening temperature.

One of the reasons for reinforcing thermoplastic polymers with glass fiber is to expand their use in technical applications that require increased strength as well as enhanced performance under load at higher temperatures. Therefore, this research also evaluated the effect of multiple material recycling of PPGF composites on the two most important thermal properties in terms of using polymer materials at elevated temperatures, namely, heat deflection temperature and Vicat softening temperature [68]. *HDT*, as a research technique, is a short-term measurement of a polymer’s resistance to applied load and allows us to determine the temperature limit of the material’s mechanical load-bearing capacity. On the other hand, the *VST* measurement technique allows us to determine the temperature at which the plastic softens and loses its stiffness [68]. Knowledge regarding the above parameters allows for the determination of the limits of applicability of polymer materials (polymers, polymer blends, polymer composites) and reflects their behavior at elevated temperatures. One of the most striking effects of the improvement in properties resulting from the addition of glass fibers in thermoplastic polymer composites is the significant increase in their heat deflection temperature [76,77]. In composites, an increase in Vicat softening temperature is also observed with an increase in GF content in the polymer, but, due to the specific nature of the *VST* measurement (point test), these changes are not comparable to *HDT* [78]. The addition of 10 wt.% GF to the PP matrix resulted in an increase in the heat deflection temperature from 56.3 ± 0.1 °C to 97.6 ± 1.1 °C (a change of 73.4%). For the composite with the highest fiber content (0PPGF30), the *HDT* value increased to 140.7 ± 0.6 °C (change of approximately 150%). In the case of the Vicat softening temperature, the highest increase occurs after reinforcing the polymer matrix with 10 wt%.% GF-*VST* increases from 153.97 ± 0.1 °C (0PP) to 161.47 ± 0.1 °C (0PPGF10). The composite containing 30 wt.% GF (0PPGF30) exhibited the highest resistance to loss of stiffness at elevated temperatures, with a *VST* value of 166.0 ± 0.2 °C. The tests confirmed that the addition of glass fiber to PP increases its mechanical load-bearing temperature limit and raises its softening temperature. Based on the changes in the dependencies shown in Figure 14, multiple mechanical recycling causes a decrease in the heat deflection temperature and the Vicat softening temperature of polypropylene and its glass fiber composites. The trend of changes in the above-mentioned thermal properties is similar to the previously described changes in strength parameters. It also results from the same mechanisms, i.e., it is caused by PP degradation processes and the effect of mechanical damage to glass fibers in PPGF composites, which occur during their repeated processing and recycling [37,38,39,40]. The lowest changes in the thermal properties under consideration, as a result of recycling five times, occurred for unfilled polypropylene and amounted to 2.8 °C for *HDT* and 1.7 °C for *VST*. Significantly higher temperature decreases, as a function of the number of recycling cycles, were observed for composites. Recycling repeated five times reduced the *HDT* value of the PPGF10 composite by 32.5%, the PPGF20 composite by 33.4% and the PPGF30 composite by 27.0%. The decreases in the softening temperatures of the composites remained similar, without regard to the degree of glass fiber filling, and they were equal to approximately. 4.0%. As in the case of mechanical properties, lower *HDT* and *VST* values than the temperatures characterizing pure polypropylene (0PP) were not obtained for any of the composite materials after five re-recycling steps.

## 4. Conclusions

The study indicated an increase in grinding efficiency, both by mass and by volume, with increasing GF content in the polymer matrix. The degradation processes that occur in the polymer, as well as the destruction of the glass fiber, were also reflected by the grinding efficiency, which was increased with an increase in the recycling multiplicity—thus contributing to a reduction in the energy input required to obtain grinding in subsequent cycles. Multiple recycling and GF content had no significant effect on the particle size distribution of the obtained regrinds. The study also showed no effect of multiple recycling on the change in bulk density of the produced regrinds. The addition of glass fiber caused changes in the luminescence of polypropylene, since its darkening was increased by increasing GF content in the composite. An unfavorable effect of glass fiber addition was also noted for the second surface property analyzed, namely, gloss. The GU value increased with the increasing composite fill rate and as a function of re-recycling steps. The recycling rate also adversely affected the hardness of the analyzed polymer materials. In contrast to the change in color and gloss, the addition of GF resulted in an improvement in the surface hardness of PP. The polymer degradation processes that occur as a result of repeated recycling, as well as the destruction of the glass fiber structure, were also reflected by the deterioration of the mechanical (static tensile and flexural, impact) and thermal (*HDT*, *VST*) properties of polypropylene and its composites. The adverse effects of multiple recycling on the glass fiber (mechanical damage due to grinding and processing, shortening of the fiber length) were confirmed by observations using optical microscopy and SEM. In the course of conducting this research, the occurrence of thermal–oxidate degradation of polypropylene due to multiple recycling and injection molding processing was also demonstrated, which was confirmed by the carbonyl index determined using the *FTIR* measurement technique. This type of degradation of PP macromolecules occurred only in composites with the highest degree of GF filling, i.e., 20 and 30 wt.%, and the *CI* value increased with each successive recycling cycle. Consideration of the issue of multiple material recycling in terms of its influence on the surface properties, the course of the grinding process and grinding characteristics of PPGF composites have not been addressed so far in the related research. Therefore, it can be concluded that the discussion regarding these issues in the presented article can be a source of technological knowledge, which can contribute to the increased use of this type of recycled composite in industrial plastics processing, with consideration of the principles of sustainable development and the circular economy.

## Figures and Tables

**Table 1:** Composition of investigated materials, density and mass flow rate (*MFR*) index.

Polymer Materials	PP	PPGF10	PPGF20	PPGF30
Glass fiber content [wt.%]	0	10	20	30
* Density [g/cm^3^]	0.895 ± 0.002	0.962 ± 0.003	1.029 ± 0.001	1.111 ± 0.002
* Bulk density [g/cm^3^]	0.550 ± 0.004	0.483 ± 0.005	0.501 ± 0.002	0.534 ± 0.002
* *MFR*_(2.16; 230)_ [g/10 min]	2.80 ± 0.02	1.98 ± 0.06	2.03 ± 0.01	2.15 ± 0.09

* own research.

**Table 2:** Glass fiber morphology after successive re-recycling steps of PPGF composites.

No. Recycle	Polymer Materials		
	PPGF10	PPGF20	PPGF30
0	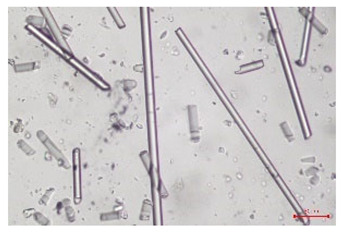	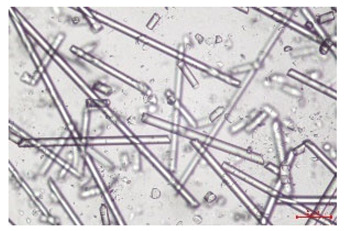	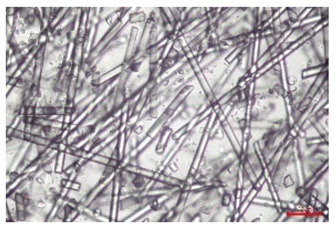
1	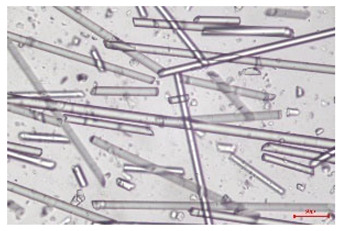	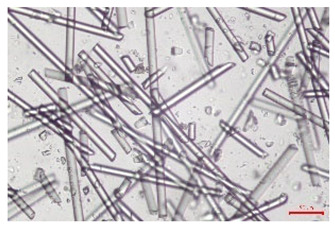	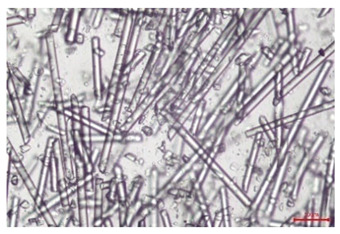
2	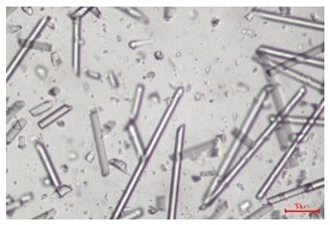	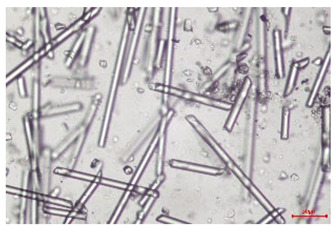	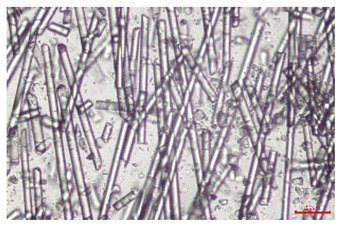
3	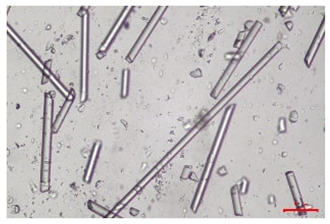	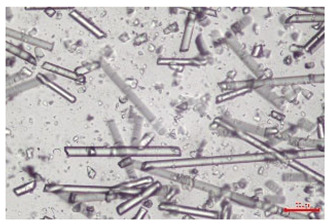	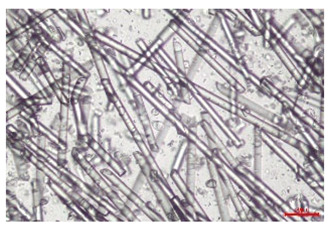
4	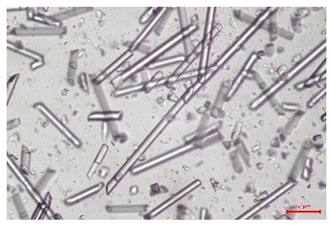	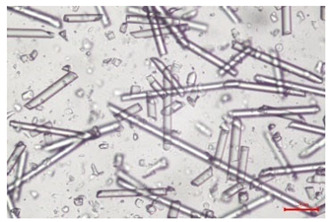	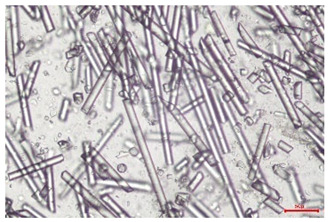
5	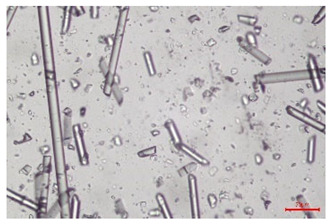	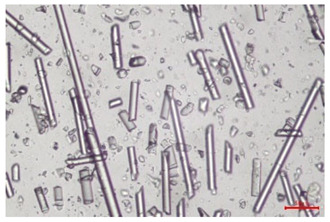	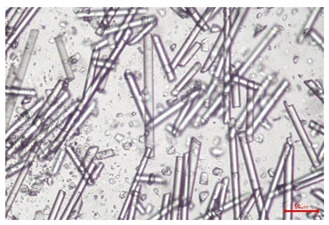

**Table 3:** Percentage of grain size distribution of PP and composites PPGF regrinds for five re-recycling cycles: sieve analysis.

Polymer Materials	Mesh Dimensions of Analytic Screens [mm]				
	7	5	4	2.5	<2.5
	Fraction Content [wt.%]				
1PP	1.07 ± 0.26	31.60 ± 1.59	17.47 ± 1.68	23.20 ± 1.22	26.66 ± 1.16
2PP	1.07 ± 0.12	32.73 ± 1.62	18.80 ± 1.22	22.27 ± 1.72	25.13 ± 1.43
3PP	1.20 ± 0.20	27.87 ± 0.81	19.53 ± 2.02	25.07 ± 0.99	26.33 ± 1.21
4PP	1.20 ± 0.20	26.33 ± 0.46	20.93 ± 1.42	24.33 ± 0.76	27.21 ± 0.87
5PP	1.00 ± 0.31	28.33 ± 0.23	20.67 ± 1.36	24.33 ± 0.50	25.67 ± 0.62
1PPGF10	0.84 ± 0.35	25.21 ± 0.76	26.47 ± 1.21	22.61 ± 0.53	24.87 ± 1.93
2PPGF10	0.67 ± 0.23	26.54 ± 0.72	25.80 ± 0.40	21.27 ± 1.60	25.72 ± 0.76
3PPGF10	0.85 ± 0.20	29.35 ± 1.20	21.87 ± 2.14	22.60 ± 1.56	25.33 ± 1.15
4PPGF10	0.87 ± 0.16	29.20 ± 0.53	22.53 ± 0.31	23.07 ± 0.31	24.33 ± 0.54
5PPGF10	0.60 ± 0.21	27.00 ± 0.35	23.20 ± 0.12	24.07 ± 0.61	25.13 ± 1.46
1PPGF20	0.43 ± 0.15	30.27 ± 1.26	21.33 ± 1.51	24.57 ± 1.44	23.40 ± 0.39
2PPGF20	0.40 ± 0.20	30.13 ± 0.95	22.13 ± 1.30	25.40 ± 1.11	21.94 ± 1.18
3PPGF20	0.33 ± 0.11	29.40 ± 1.39	20.80 ± 0.53	25.13 ± 1.67	24.34 ± 0.84
4PPGF20	0.40 ± 0.18	27.80 ± 2.46	21.80 ± 0.53	26.67 ± 1.62	23.33 ± 1.97
5PPGF20	0.53 ± 0.12	28.73 ± 1.36	22.73 ± 2.00	23.47 ± 0.46	24.54 ± 0.51
1PPGF30	0.33 ± 0.17	35.00 ± 1.25	22.67 ± 0.76	25.53 ± 0.31	16.47 ± 0.46
2PPGF30	0.13 ± 0.12	34.00 ± 1.51	23.73 ± 1.33	23.40 ± 1.46	18.74 ± 0.66
3PPGF30	0.33 ± 0.12	32.07 ± 0.31	25.87 ± 1.62	24.33 ± 0.50	17.40 ± 1.12
4PPGF30	0.33 ± 0.10	31.00 ± 1.00	25.40 ± 1.59	26.67 ± 1.67	16.60 ± 0.74
5PPGF30	0.07 ± 0.11	27.33 ± 1.27	26.20 ± 0.72	28.60 ± 1.06	17.80 ± 0.28

**Table 4:** IR absorption bands of polypropylene [70].

Wave Number, cm^−1^	Absorbing Group and Type of Vibration
2916	νa (CH_2_)
2959	νa (CH_3_)
2881	νs (CH_3_)
2841	νs (CH_2_)
1460	δa (CH_3_)
1376	δs (CH_3_)
1357	γw (CH_2_-CH)
1328	γw (CH_2_-CH)
1302, 1224, 941	Carbon lattice pulsation
1170, 1153	γw (CH_3_), δ (CH_2_), δ (CH)
975, 899	γr (CH_3_), νr (CH_2_), νr (CH)
841, 810	γr (CH_2_), νr (CH), νr (CH_3_)
765	γw (CH_2_)

## Data Availability

The original contributions presented in this study are included in the article. Further inquiries can be directed to the corresponding author.
